# Protection of Rat Cardiac Myocytes by Fructose-1,6-Bisphosphate and 2,3-Butanedione

**DOI:** 10.1371/journal.pone.0035023

**Published:** 2012-04-27

**Authors:** Thomas J. Wheeler, Sufan Chien

**Affiliations:** 1 Department of Biochemistry and Molecular Biology, University of Louisville School of Medicine, Louisville, Kentucky, United States of America; 2 Department of Surgery, University of Louisville School of Medicine, Louisville, Kentucky, United States of America; Medical College of Georgia, United States of America

## Abstract

Earlier studies by our group showed that fructose-1,6-bisphosphate (FBP) enhances the hypothermic preservation of rat cardiac myocytes and the functional recovery of animal hearts after hypothermic storage. However, the mechanisms involved were not clear. We extended the cardiomyocyte studies by testing whether the FBP effects were due to chelation of extracellular calcium, leading to lower intracellular levels. We also tested effects of 2,3-butanedione monoxime (BDM), pyruvate, and adenine nucleotide precursors. Cardiomyocytes were incubated in ischemic suspension at 3°C, and aliquots examined over 48 to 72 hours for retention of rod-shaped morphology, a measure of viability. Cytosolic Ca^2+^ levels were measured in some experiments. FBP at 5 mM reduced the death rate even when added after one or two days of incubation. It caused cytosolic calcium levels that were 33% lower than controls in freshly-isolated cells and 70% lower after one day of incubation. EGTA protected against cell death similarly to FBP. These results indicated that one of the mechanisms by which FBP exerts protective effects is through chelation of extracellular calcium. BDM was strongly protective and reduced cytosolic calcium by 30% after one day of incubation. As with FBP, BDM was effective when added after one or two days of incubation. BDM may be useful in combination with FBP in preserving heart tissue. Pyruvate, adenine, and ribose provided little or no protection during hypothermia.

## Introduction

Heart transplantation is a life saving procedure for patients with end-stage heart failure. However, techniques for heart preservation have changed very little over decades [1]. Current hypothermic preservation is still limited to 4–6 hours, not much better than what was obtained five decades ago. To increase myocardial survival times, various additives have been proposed [2], but the results have not been conclusive. Among these additives, fructose-1,6-bisphosphate (FBP), 2,3-butanedione monoxime (BDM), pyruvate, adenosine, ribose, and adenine have all been reported to have some effects. Of special interest is FBP, which has been reported to be useful in protecting a variety of tissues during ischemia and hypoxia. These include heart (e.g., Ref. [3]), liver [4], kidney [5], brain [6], smooth muscle [7], lung [8], and intestine [9]. New studies concerning benefits of FBP appear every year (e.g., [10]–[13]). Our group has characterized effects of FBP in preserving heart function during hypothermic storage [3], [14]–[15], and has demonstrated uptake of FBP by cardiac myocytes [16]–[17], even at 3°C [17]. We also showed that in an experimental model for hypothermic heart preservation, isolated cardiac myocytes maintained in ischemic suspension at 3°C, FBP greatly reduced the death rate (as measured by loss of rod-shaped morphology) and helped preserve cellular ATP [18]. In other papers concerning use of FBP with the heart, the compound has been included in the preservation solution in a study of continuous perfusion during cold storage [19], and in clinical trials of coronary artery bypass graft surgery [20]–[21].

Several hypotheses have been proposed for the mechanism by which FBP protects tissues. One possibility is that FBP enters cells and is used in glycolysis, providing ATP without the necessity of the two prior ATP-consuming phosphorylation steps. Another is that FBP exerts its effects via chelation of calcium ions [22]. Other proposals include allosteric activation of phosphofructokinase and stimulation of the pentose phosphate pathway. However, none of the proposals have been definitely established.

Hassinen et al. [22] determined a value of about 3 mM for the dissociation constant of the Ca^2+^-FBP complex. Thus, millimolar levels of FBP, as used in our previous experiments [18], could reduce extracellular levels of Ca^2+^, which in turn would allow the myocytes to maintain their intracellular Ca^2+^ at lower levels and reduce the amount of ATP consumed by Ca^2+^ transport.

The work described here focused on several additives that have been reported to be effective in myocardial protection. We paid special attention to the calcium chelation hypothesis for FBP effects, again using isolated cardiac myocytes as an experimental system. Our results indicate that chelation of extracellular calcium is an important potential mechanism by which FBP protects cells. We also tested whether 2,3-butanedione monoxime (BDM) and pyruvate, both of which have shown protective effects with intact heart and with cardiac myocytes, would be beneficial in our experimental system. BDM was strongly protective, while pyruvate had little effect. Finally, we tested the hypothesis that adenine and ribose, either individually or in combination, could enhance the survival of the myocytes due to their ability to serve as precursors for adenine nucleotides. The results did not support this hypothesis.

## Materials and Methods

### Animals

Animal protocols were approved by the Institutional Animal Care and Use Committee of the University of Louisville (Proposal 04152).

### Reagents

Collagenase was from Worthington Biochemical Corporation (Lakewood, NJ) (collagenase type II) or from Roche Applied Science (Indianapolis, IN) (Liberase Blendzyme 2). Albumin, ethylenediaminetetraacetic acid (EDTA), ethylene glycol-bis(2-aminoethylether)-N,N,N′,N′-tetraacetic acid (EGTA), 2,3-butanedione monoxime (BDM), pyruvic acid, and ribose were from Sigma Chemical Co. (St. Louis, MO). Fructose-1,6-bisphosphate, trisodium salt, was a gift from Paul J. Marangos, PhD, of Cypros Pharmaceutical Co. (Carlsbad, CA). Fura-2AM was from Molecular Probes (Eugene, OR) and adenine from ICN Biomedicals (Aurora, OH).

### Preparation of Cardiac Myocytes

Quiescent, calcium-tolerant cardiac myocytes (typically about 70–80% rod-shaped) were prepared by a modification [23] of the method of Fischer et al. [24] using male Sprague-Dawley rats, as in our previous study [18]. The final washes and cell suspensions were performed using medium E of Fischer et al. [24], consisting of 6 mM KCl, 1 mM Na_2_HPO_4_, 0.2 mM NaH_2_PO_4_, 1.4 mM MgSO_4_, 128 mM NaCl, 10 mM sodium (4-(2-hydroxyethyl)-1-piperazineethanesulfonic acid (HEPES), pH 7.4, and 2% fatty acid-free albumin.

### Assay of Myocyte Preservation

Hypothermic preservation of cardiac myocytes was assayed as before [18]. Retention of rod-shaped morphology was used as a measure of viability [25]. Briefly, washed cells were suspended to concentrations of about 2 to 3×10^5^/ml in medium E (see above) plus other compounds being tested. Each compound (or the combination of adenine plus ribose) other than FBP was tested both in the presence and absence of 5 mM FBP within the same experiment, and each experiment included two controls (no additions) plus one sample with 5 mM FBP tested alone. In most experiments, eight aliquots of 100 μl for each test condition were placed in microcentrifuge tubes (0.5 ml) and incubated in a refrigerator (3°C); these were resuspended and counted at various time points over a 72 hour period. For the experiments with BDM, five aliquots of 50 μl for each condition were counted at various time points over 48 hours. At each time point, an aliquot was resuspended and two separate samples taken for counting with a hemacytometer, with total and rod-shaped cell numbers recorded. Samples were blinded prior to counting (except for the experiments in which FBP or BDM were added after one or two days of hypothermic incubation). The data were fitted by nonlinear regression (using either GraphPad Prism 4 or the Solver feature of Microsoft Excel) to the equation for first-order decay:
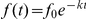
where *f*(*t*) is the fraction of rod-shaped cells at time *t*, *f*_0_ the fraction at time zero, and *k* the first-order rate constant. The rate constant was then compared to the average of the rate constants for the two control incubations within the same experiment (given the same hypothermic incubation but with no additions to the medium) to obtain a relative death rate. These relative rates were then averaged over all experiments to give the values presented in the figures.

### Assays of Intracellular Calcium

Intracellular calcium levels were measured by a modification of the procedures of Wan and Dean [26]. Cells previously incubated in medium with or without 5 mM FBP or 5 mM BDM were concentrated to 10^6^ cells/ml in the same medium plus 1 μg/ml fura-2AM. After a 30 min incubation, the cells were washed 3 times with medium (with the same concentration of FBP or BDM) and diluted to 1.25×10^5^ cells/ml. Fluorescence was measured in a Perkin Elmer LS50B Luminescence Spectrometer with FL WinLab software (version 3). Excitation alternated between 340 and 380 nm, while emission was measured at 510 nm. After obtaining a stable sample reading, 20 μM digitonin and 1 mM CaCl_2_ were added to obtain the maximal Ca^2+^ signal, followed by addition of 50 mM EGTA to determine the minimal signal. Calcium concentrations were calculated from the emission data using the Grynkiewicz equation [27] as described [26].

### Assays of Calcium in Media

Calcium concentrations in media were estimated by two different methods. In the first, fluorescence of diluted samples was measured using the Fura-2 Calcium Imaging Calibration Kit from Invitrogen. A calibration curve was constructed using the InCyt Im2 software. In the second, atomic absorption was measured using the Perkin Elmer AAnalyst 100 Spectrometer. A diluted sample (1.8 ml) was mixed with 0.9 ml of 40% nitric acid and 0.3 ml of LaCl_3_ (0.1 g/ml). This was centrifuged (44×g for 10 min) to precipitate the protein. The supernatant was removed and subjected to a second centrifugation prior to making the atomic absorption measurement. A calibration curve was constructed using Ca^2+^ samples of known concentration.

### Statistical Analysis of Data

Calculations were performed using the QuickCalcs site of GraphPad Software (http://graphpad.com/quickcalcs/index.cfm). Paired t-tests using the individual rates were employed for analyzing death rates. A sign test was used for analyzing the effect of FBP on cytosolic calcium.

## Results

### Effects of FBP Addition After One or Two Days of Hypothermic Incubation

**Figure 1:**
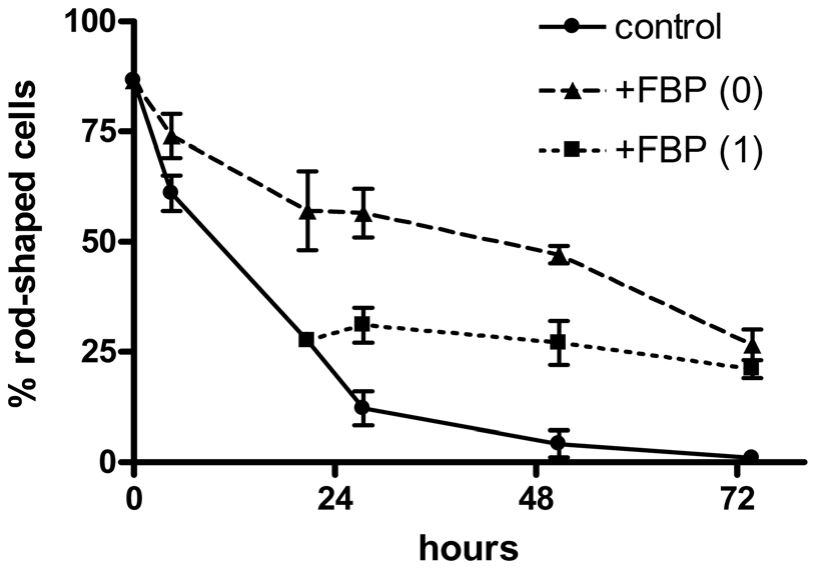
Effects of FBP addition after one day of hypothermic incubation. Cardiac myocytes were incubated at 3°C for the time indicated on the x-axis, with additions of 5 mM fructose-1,6-bisphosphate prior to the beginning of the incubation (triangles) or after one day (squares); controls (circles) had no addition. The percentage of rod-shaped cells at each time point is shown. Results are means from two experiments. Error bars indicate the individual values (in some cases the bars lie within the symbols).

In our previous study [18], we observed that fructose-1,6-bisphosphate greatly reduced the loss of viability (as indicated by rod-shaped morphology) in rat cardiac myocytes kept in ischemic suspension at 3°C. Retention of rod-shaped morphology is highly correlated with dye exclusion, a common measure of viability [25]. The optimum concentration of FBP was about 5 mM, which reduced the death rate by about 65%. In these experiments the FBP was added to the cells immediately before the cells were placed in a refrigerator. We were interested in whether the protective effect of FBP required its presence during the transition from room temperature to 3°C, or whether it would be protective even if added later. In the experiments shown in Fig. 1, myocytes were initially incubated with (triangles) or without (circles) 5 mM FBP. As was typically seen, FBP greatly reduced the loss of rod-shaped morphology over the next 72 hours. When FBP was added beginning after 24 hours of incubation (squares), little or no loss of rod-shaped morphology was observed over the next 48 hours, compared to the control cells, which continued to change their morphology. In one of the experiments, FBP was also added after 48 hours of incubation, and the morphology was preserved over the next 24 hours (data not shown).

These results suggested either that FBP could be taken up and metabolized, giving protective effects, even at 3°C; or that it exerted its protective effects through other mechanisms not requiring metabolism. One such mechanism that has been proposed is chelation of calcium [22].

### Effects of Calcium and Chelating Agents on Myocyte Preservation

**Figure 2:**
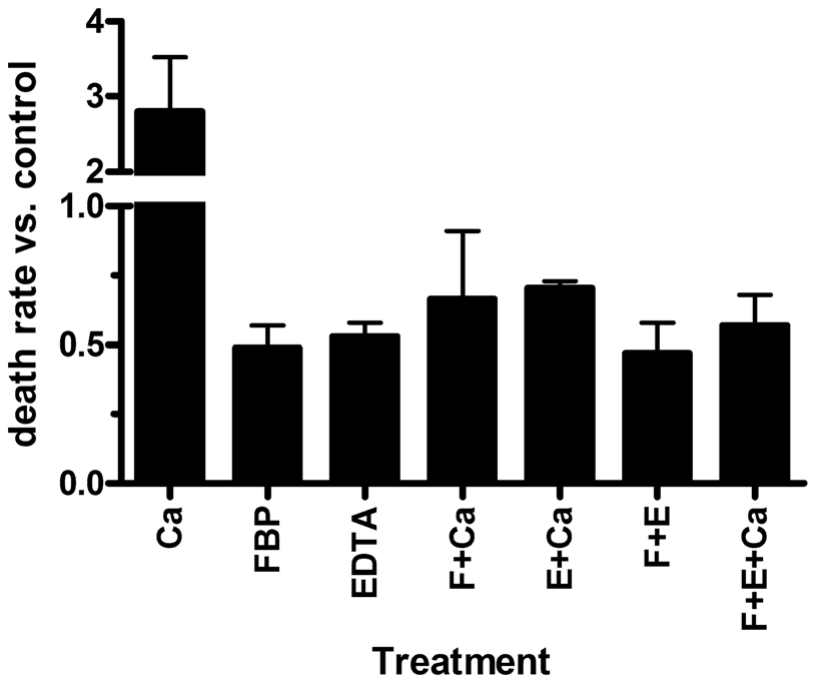
Effects of calcium and EDTA on hypothermic preservation of cardiac myocytes. Cells were incubated with no additions or with 100 μM calcium, 5 mM FBP, 0.5 mM EDTA, or various combinations of additions. Death rates for treated cells were normalized to those of control cells. The averages of the relative death rates for two experiments are plotted, with error bars indicating S.E.M.

The medium used for the hypothermic preservation experiments, medium E (see above, “Preparation of Cardiac Myocytes”), contains no calcium other than that contributed by the albumin. A test of the effects of additional calcium indicated that levels up to 10 μM had little effect, but levels of 30 μM and higher greatly increased the death rate (data not shown). We performed two experiments in which 100 μM calcium was added, with or without FBP (5 mM) or EDTA (0.5 mM). Results are shown in Fig. 2. The added calcium more than doubled the death rate (first bar). However, in the presence 100 μM calcium and either FBP (4th bar) or EDTA (5th bar), the death rate was lower than in control cells (no additions), and was nearly as low as with FBP alone (2nd bar). EDTA alone (3rd bar) also protected similarly to FBP alone. Thus, either FBP or EDTA can overcome the damaging effects of a large increase in the medium calcium level.

**Figure 3:**
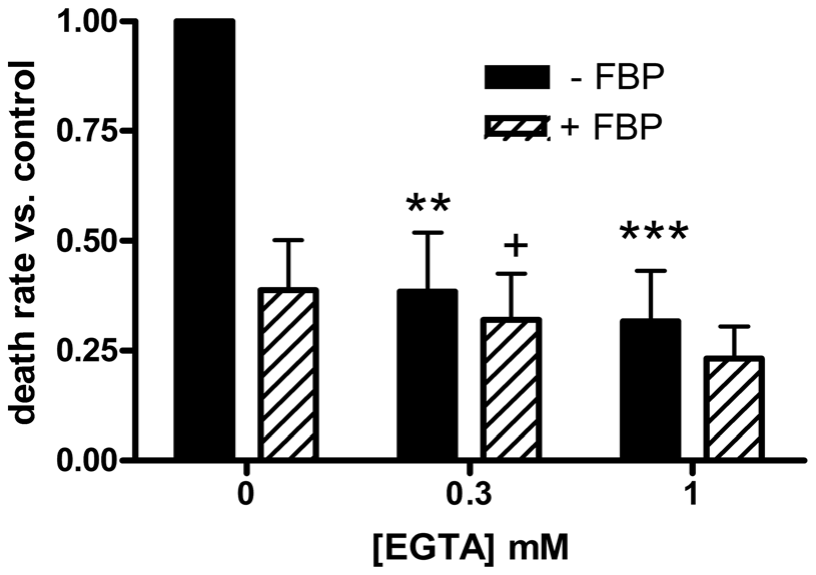
Effects of EGTA on hypothermic preservation. In this figure and in Figure 5, death rates for treated cells were normalized to those of control cells in the same experiment, and the relative death rates averaged over all experiments. Filled bars in these figures are for treatments without FBP, while hatched bars are for treatments including 5 mM FBP. Results are means of 4 determinations; error bars indicate S.E.M. In this figure, myocytes were incubated with the indicated concentrations of EGTA. Statistically significant by paired t-tests: **, p<0.01 vs. control; ***, p<0.005 vs. control; +, p<0.05 vs. FBP alone.

Additional experiments were performed using EGTA, which is more specific for calcium chelation. We measured preservation of myocytes in the presence or absence of EGTA (both with and without 5 mM FBP) (Fig. 3). EGTA alone (solid bars) reduced the death rate by about 60% at 0.3 mM and 70% at 1.0 mM. It appeared that the combination of EGTA and FBP (hatched bars) offered greater protection than FBP alone (furthest left hatched bar). Paired t-tests gave p = 0.040 for 0.3 mM EGTA plus FBP vs. FBP alone and p = 0.066 for 1 mM EGTA plus FBP vs. FBP alone. A 77% reduction in the death rate was observed with 1 mM EGTA plus FBP.

### Effects of FBP on Cytosolic Calcium Levels

**Figure 4:**
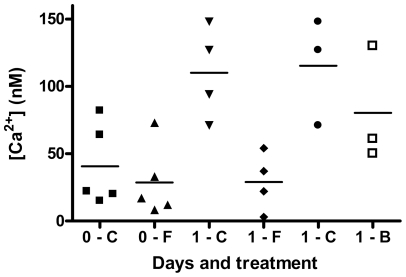
Effects of FBP and BDM on intracellular calcium. Aliquots of myocytes were given no additions, 5 mM FBP, or 5 mM BDM, and analyzed for intracellular Ca2+ either immediately or after 24 hours of incubation at 3°C. Individual results are plotted, with means of the groups indicated by horizontal lines. The first two sets of data are for five experiments with fresh myocytes without (▪) or with (▴) FBP. The second two sets of data are for four experiments with myocytes incubated for one day without (▾) or with (♦) FBP. The last two sets of data are for three experiments with myocytes incubated for one day without (•) or with (□) BDM.

In order to test whether the effects of FBP on preservation were related to intracellular calcium levels, we incubated freshly prepared myocytes with or without 5 mM FBP plus the cell-permeable Ca^2+^ indicator fura-2AM (which is cleaved to the impermeable fura-2 inside the cell). The cells were then washed and the fluorescence measured to determine the cytosolic free Ca^2+^ levels. Results of five experiments are shown in the first two data sets of Fig. 4, which plots the individual results. While the raw values differ considerably among experiments, in all five experiments the FBP-treated cells (upward pointing triangles) had lower calcium levels than the controls (filled squares) (p<0.05 by sign test). When the ratio (FBP-treated vs. control) within each experiment was averaged over all experiments, a reduction in calcium of 33±7% was seen.

We also examined calcium levels in myocytes that had been incubated at 3°C for 24 h with or without 5 mM FBP (Fig. 4, third and fourth sets of data). Again, the calcium levels were in every case lower (by an average of 70±11%, p<0.005) for FBP-treated cells (diamonds) than for controls (downward pointing triangles). The mean concentrations were 29 nM for FBP-treated cells vs. 110 nM for controls.

### Calcium Levels in Media

The similarity of the protective effects of FBP and EGTA supports the role of chelation of extracellular Ca^2+^ in the preservation, even though the final medium (medium E) is prepared without calcium. We attempted to measure the levels of Ca^2+^ typically present in our incubation medium by two different methods. Our survival experiments were performed with a constant level of albumin (2%). However, to assess possible contributions of the albumin to calcium in the media we tested samples with 1% or 3% albumin. We first employed a fluorescence method using a fura-2 calcium imaging calibration kit. Various aliquots of the media gave calculated concentrations of 1 to 8 μM Ca^2+^ for the undiluted media, and the levels were higher for media with 3% albumin compared to those with 1% albumin. However, the calculated original concentrations were higher when larger aliquots were assayed, so the actual values could not be determined. The second method was atomic absorption. For these a sample of medium without albumin was also tested. These measurements gave much higher calculated concentrations (50 to 200 μM Ca^2+^) for the undiluted albumin-containing media, but these samples had to be centrifuged twice to remove precipitates, and possibly some residual precipitated material gave anomalously high readings. Nevertheless, the results agreed with the fluorescence method insofar as 3% albumin gave higher readings than 1% albumin. A sample of albumin-free medium gave a calculated concentration of 0.3 μM, indicating that the albumin is the major source of calcium in the incubation medium.

### Effects of BDM on Preservation and Cytosolic Calcium

**Figure 5:**
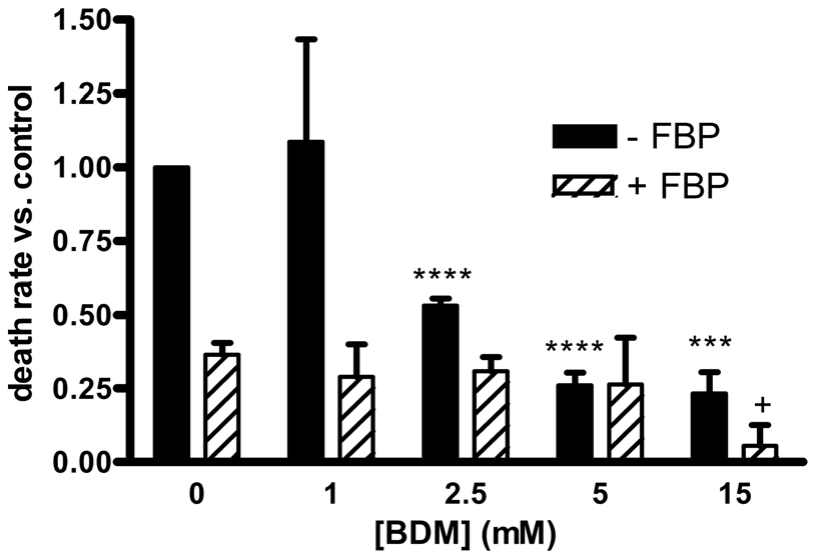
Effects of 2,3-butanedione monoxime on preservation. Myocytes were incubated with the indicated concentrations of BDM with or without 5 mM FBP. Results are means of 3 determinations. Statistically significant by paired t-tests: ***, p<0.005 vs. control; ****, p<0.002 vs. control; +, p<0.05 vs. FBP alone.

**Figure 6:**
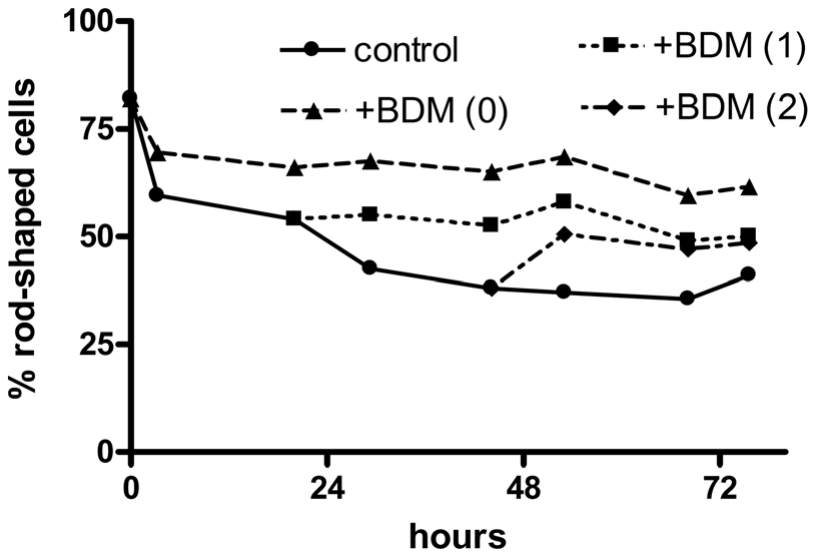
Effects of BDM addition after one or two days of hypothermic incubation. Cardiac myocytes were incubated at 3°C for the time indicated on the x-axis, with additions of 5 mM 2,3-butanedione monoxime prior to the beginning of the incubation (triangles) or after one (squares) or two (diamonds) days; controls (circles) had no addition. The percentage of rod-shaped cells at each time point is shown. Results are means from two experiments.

Fig. 5 shows the effects of 1 to 15 mM 2,3-butanedione monoxime, in the presence and absence of 5 mM FBP, on the hypothermic preservation. At 2.5 mM and higher, BDM alone (solid bars) greatly decreased the death rate (p<0.005 for 15 mM and P<0.002 for 2.5 and 5 mM); the decrease was about 75% at 5 and 15 mM. Moreover, the combination of 15 mM BDM plus 5 mM FBP (hatched bar at far right) produced a decrease of 94 ± 7%, which was significantly greater (p<0.05) than the effect of FBP alone (furthest left hatched bar). We tested whether the effects of BDM required that it be added at the initiation of the incubation, performing experiments similar to those done with FBP (Fig. 1). The results (Fig. 6) show that addition of BDM even after one or two days of hypothermic incubation was protective. (Although it appears that the addition of BDM after two days resulted in an increase in the fraction of rod-shaped cells, this merely reflects the variation in counts between different aliquots. A given aliquot, once counted, was not used further.).

We also examined the effect of BDM on cytosolic calcium levels after 24 h of hypothermic preservation (Fig. 4, fifth and sixth sets of data). In three experiments, BDM (5 mM) (open squares) reduced the calcium levels by 29±18% compared to cells incubated for the same time without BDM (circles). This was a much smaller effect than observed with 24 hours of FBP treatment (Fig. 4, diamonds vs. downward pointing triangles), even though the effect of 5 mM BDM on preservation appeared somewhat greater than that of 5 mM FBP (Fig. 5, fourth solid bar compared to first hatched bar).

### Effects of Pyruvate, Adenine, and Ribose on Myocyte Preservation

In other experiments testing potential metabolic interventions for hypothermic preservation, we examined effects of pyruvate, adenine, and ribose. Pyruvate was tested at 2, 5, and 10 mM, in both the presence and absence of 5 mM FBP (data not shown). In the absence of FBP, pyruvate appeared to produce modest protective effects (10–20% decrease in the death rate). However, the differences from control were not statistically significant. In the presence of FBP, no benefits were observed beyond those of FBP alone.

In order to test whether the adenine nucleotide precursors adenine and ribose could enhance preservation, we studied the effects of these two compounds (0.5 to 10 mM) individually; in equimolar combination with each other; and in combination with 5 mM FBP (data not shown). No significant protective effects were observed for adenine and/or ribose without FBP, and the compounds did not appear to enhance the protective effects of FBP. The combination of 10 mM adenine plus 10 mM ribose appeared to increase the death rate, though the effects were not statistically significant.

## Discussion

### Possible Protective Effects of FBP Due to Chelation of Calcium

We observed that 5 mM FBP could protect myocytes from death during ischemic, hypothermic incubation even if added after one day (Fig. 1) or two days. This suggested that a mechanism other than metabolism of FBP might be involved in its protective effects, with chelation of extracellular calcium by FBP being a possibility [22]. Although the myocytes are referred to as "calcium tolerant" because they survived increasing amounts of calcium during the isolation procedure [24], the final preparation nevertheless shows reduced viability during hypothermic incubation with added calcium (Figure 2). Likely this is because increasing extracellular calcium will lead to a rise in intracellular calcium. The latter can be damaging via several mechanisms, including expenditure of ATP for Ca^2+^ATPases, as the cells attempt to lower their cytosolic calcium levels; activation of contractile activity, also reducing ATP; and activation of Ca^2+^-dependent proteinases. By reducing the extracellular Ca^2+^ level, FBP would make it easier for cells to maintain low intracellular levels and prevent such damage.

In the experiments shown in Fig. 3, the calcium-chelating agent EGTA reduced the death rate by 60–70%, similar to the effects of FBP. Thus, chelation of calcium is protective in our experimental system, even though the final medium used to wash and incubate the myocytes (medium E) is nominally calcium-free. It is difficult to measure the levels of calcium in this medium. However, the results indicated that the medium likely contained micromolar levels of calcium, and that most of this probably came from the albumin. Thus, the idea of a chelating effect of extracellular FBP is reasonable. Using a dissociation constant of 3 mM [22], 5 mM FBP would bind approximately 60% of the calcium in the medium. We found that there was considerable variation in the quality of myocytes prepared using different lots of albumin having the same product number. Possibly this was the result of differences in calcium content of the different batches.

Evidence for effects of FBP on calcium homeostasis is shown in Fig. 4. For freshly-prepared myocytes (first two sets of data), cytosolic calcium was an average of 33% lower in FBP-treated cells (upward pointing triangles) compared to control cells (solid squares). For freshly-prepared cells there is expected to be little effect of FBP on the cellular ATP levels; in Ref. [18] we observed only a 16% higher ATP level for cells treated with 5 mM FBP compared to control cells after 2 hours of hypothermic incubation. Thus, the differences for the fresh myocytes in Fig. 4 are likely direct effects of calcium chelation rather than due to increased availability of ATP for Ca^2+^ pumping. Our earlier experiments [18] found that ATP levels were about 30% higher at 6 hours and 50% higher at 24 hours in FBP-treated cells. Therefore the larger reduction in calcium with 24 hours of FBP treatment (Fig. 4, diamonds vs. downward pointing triangles) may be due to both chelation of Ca^2+^ by FBP and the cumulative effects of maintaining higher ATP levels in the cells, which should allow them to reduce the calcium levels through Ca^2+^-ATPase activity.

In addition to producing extracellular effects, it is possible that FBP could be taken up by the cells and chelate intracellular Ca^2+^. We previously showed that label from radiolabeled FBP at 5 mM could be taken up by myocytes, both at room temperature and at 3°C [15]. However, it is likely that much of this label was converted to other metabolites, and it seems unlikely that intracellular FBP would rise to levels high enough to provide a significant chelating effect.

Additional support for a chelating effect of extracellular FBP, rather than an effect via glycolytic ATP production, comes from a comparison of the effects of BDM and FBP. These compounds at 5 mM had similar effects on the hypothermic survival of myocytes (Fig. 5, first hatched bar vs. fourth filled bar). However, in myocytes incubated for 24 hours, BDM (which could affect calcium levels indirectly by preserving ATP) produced a much smaller reduction in free calcium (Fig. 4, open squares vs. circles) than did FBP (Fig. 4, diamonds vs. downward pointing triangles).

Nevertheless, the data in Fig. 3 indicate that FBP may have protective effects beyond those due to calcium chelation. For both 0.3 and 1.0 mM EGTA, it appeared that the combination of FBP and EGTA (hatched bars) produced greater reductions in the death rate than EGTA alone (solid bars), although the differences were not statistically significant at the p<0.05 level by paired t-tests (p = 0.06 and 0.12 for 0.3 and 1.0 mM EGTA, respectively). Because of the much greater affinity of EGTA for Ca^2+^ compared to the affinity of FBP, 5 mM FBP would not lower the Ca^2+^ level significantly in the presence of these levels of EGTA. We previously showed that FBP could be taken up by cardiac myocytes at 21°C and 3°C [17]. It is possible that FBP taken up during or after the transition to hypothermia could be used to provide glycolytic ATP (without the need for the ATP-consuming steps in glycolysis) even at the reduced temperature. Since energy-consuming processes would also be slowed by the hypothermia, this ATP could provide significant protection against cell death. As described above, we previously demonstrated that FBP helped maintain higher levels of ATP during hypothermic incubation [18]. It should be noted that Hassinen et al. [22] reported metabolic effects of FBP in perfused hearts that were not produced by EGTA and thus did not appear to be due to calcium chelation.

### Previous Studies of FBP and Calcium

Effects on Ca^2+^ levels are often mentioned as a possible mechanism of FBP action. Several studies (e.g., Ref. [22]) have shown reduction in Ca^2+^ in extracellular media in the presence of FBP, but their conclusions differ. Only a few papers have actually measured changes in intracellular Ca^2+^ concentrations due to FBP. Bickler and Kelleher [28] observed that FBP prevented hypoxia-induced increases in intracellular Ca^2+^ in brain slices and astrocytes. Effects on basal levels were not reported. While the authors suggested that preservation of ATP was responsible for the FBP effect, they also suggested that Ca^2+^ chelation could be involved. Cavallini et al. [29] showed that FBP inhibited the thrombin-induced increase in cytosolic Ca^2+^ in platelets, though it did not appear that there was any effect on the resting Ca^2+^ level. The authors proposed effects on “the transmission of signal at the level of the receptor-G-protein-phospholipase C system.” Tamaki et al. [30] reported that FBP inhibited the increase in cytosolic Ca^2+^ in response to phorbol ester treatment of Kupffer cells (data were reported as fluorescence recordings rather than Ca^2+^ concentrations). The authors proposed that the effects came about via both chelation of extracellular Ca^2+^ and by provision of glycolytic ATP, allowing greater Ca^2+^-ATPase activity. Two of the studies that looked at intracellular Ca^2+^ [31]–[32] concerned neurons, and found increases in cytosolic Ca^2+^ in response to FBP treatment, in contrast to our observations. However, in Ref. [31], FBP, while it increased basal Ca^2+^ levels, prevented the increase in Ca^2+^ in response to hypoxia. Although chelation of Ca^2+^ was mentioned as possibly contributing to this effect, the authors concluded that “chelation does not account for the entirety of FBP's protective properties,” and they favored a mechanism involving phospholipase C signaling.

In another study, using synaptosomes rather than intact cells, Zeng et al. [33] showed that FBP reduced the free Ca^2+^ level during ischemic conditions. They proposed metabolic effects of FBP and did not address the possible role of chelation.

Thus, our studies add to the weight of evidence concerning a role for Ca^2+^ in protective effects of FBP, and provide the first experimental evidence related to an effect of FBP on Ca^2+^ homeostasis in heart preservation. Possible further experiments that would help confirm our hypothesis would include determining the effect of FBP on survival under conditions in which extracellular Ca^2+^ is fixed (using solutions of Ca^2+^ and EGTA), as well as measurements of intracellular Ca^2+^ (similar to those in Fig. 4) for EGTA-treated myocytes. Determining the combined effect of EGTA and FBP on intracellular Ca^2+^ would help establish whether or not FBP acts by multiple mechanisms.

### Effects of BDM

2,3-Butanedione monoxime (BDM) has several effects in the heart [34]. These include effects on calcium fluxes at low concentrations and inhibition of myosin ATPase at somewhat higher concentrations. Beneficial effects of BDM in the preparation of cardiac myocytes have been characterized [35], and the compound is used in the procedure we employed [24]. Several studies (e.g., Ref. [36]) have found benefits in preservation of the intact heart. However, at high concentrations, BDM can also have deleterious effects, possibly through action as a phosphatase [34].

We found that BDM produced a marked protective effect (comparable to that of FBP) during hypothermic incubation of myocytes (Fig. 5). As was the case for FBP, BDM reduced the death rate even when added after one or two days of hypothermic incubation (Fig. 6). Treatment with BDM for 24 hours also resulted in a lower cytosolic calcium concentration (Fig. 4, open squares) compared to untreated cells (circles). However, the effect was much lower than that of FBP (Fig. 4, diamonds vs. downward pointing triangles). Possibly this is because the effect of BDM on calcium is indirect; by inhibiting myosin ATPase and preserving ATP, it enables the cells to maintain calcium pumping activity better than untreated cells. Because they have different modes of action, the combination of FBP and BDM may have benefits beyond either alone, as indicated by the furthest right hatched bar in Fig. 5.

Kivistö et al. [37] observed protective effects of BDM (25 mM) on cardiac myocytes incubated for 24 hours at 5°C, in agreement with our results. However, these experiments used myocytes deliberately isolated under “stressed” conditions, such that less than 1% to 10% of the cells remained viable after 24 hours with the various treatments. By comparison, in our studies with BDM, control cells averaged 41% viability after 24 hours, while those treated with 1 to 15 mM BDM averaged 56% viability.

### Effects of Pyruvate, Adenine and Ribose

Pyruvate is employed during the procedure we used for the preparation of cardiac myocytes [24]. The general metabolic benefits of pyruvate in the heart have been reviewed by Mallet [38]. Most studies of pyruvate's effects on the heart have examined periods of reperfusion after ischemia, rather than effects during cold storage. We found that pyruvate produced, at best, relatively small decreases in the death rate of myocytes incubated at 3°C. When combined with 5 mM FBP, effects were no greater than those of FBP alone. While we hypothesized that there might be sufficient residual oxygen in the ischemic cell suspensions to support pyruvate oxidation, the results suggest that either such metabolism is relatively small, or it provides little survival advantage to the myocytes. This is consistent with our previous finding that dichloroacetate, which stimulates pyruvate dehydrogenase, produced no beneficial effects under these conditions [18].

The critical role of maintaining ATP levels in the survival of the heart during cold storage has long been recognized [39]. Even if glycolysis is enhanced (e.g., by provision of FBP), the supply of adenine nucleotides could limit energy production. In addition to conversion of ATP to ADP and AMP, the total adenine nucleotide pool can be depleted under ischemic conditions due to the further degradation to adenosine, inosine, and hypoxanthine, all of which can penetrate the plasma membrane and be lost to cardiac myocytes [40]. One strategy for overcoming this problem is to provide the precursors for de novo adenine nucleotide synthesis, adenine and ribose. In various studies, these two compounds have been shown to be beneficial for the heart, either alone or in combination. However, these experiments have focused on recovery of function and of ATP during reperfusion, rather than preservation during a period of cold storage.

We tested whether adenine and ribose, when present during hypothermic incubation of cardiac myocytes, could reduce their death rates. Neither adenine nor ribose showed significant effects when added individually. Moreover, the combination of the two also was not beneficial. While it is possible that the treatments would aid in the resynthesis of adenine nucleotides following return to normoxia and normothermia, it appeared that any synthesis of adenine nucleotides was insufficient to improve survival while the cells remained under ischemic, hypothermic conditions.

### Conclusions

The results presented here extend our earlier work on the ability of FBP to preserve cardiac myocytes under hypothermic conditions [18]. Our findings indicate that one major effect of FBP is its chelation of extracellular Ca^2+^, in addition to other mechanisms. An appreciation of the ability of FBP to chelate Ca^2+^, and thus affect intracellular calcium levels, is of importance because FBP has been used for preservation of a wide variety of types of tissues [3]–[9], as well as in many other types of experiments. We used an extreme endpoint for assessing the effects of FBP (protection against cell death), but it is likely that FBP would help protect cell functions even in cases where little or no cell death is occurring.

Recent studies have often focused on diverse pathways that are altered in response to FBP, such as inflammation [41] and apoptosis [42]. However, the underlying connection between FBP and such effects are unclear. Our results suggest two linked mechanisms for FBP effects: calcium and energy. If FBP acts to chelate calcium, it will spare ATP that would otherwise be used in calcium pumping. Alternatively, If FBP is used to provide glycolytic ATP, the increased cellular energy can help control intracellular Ca^2+^ levels. Together, effects of FBP on ATP and Ca^2+^ levels will influence many regulatory pathways, and these pathways deserve further exploration.

Also, in the course of our studies we observed that albumin can contribute significant amounts of calcium to cell culture media, and that different lots of the same commercial albumin product appear to differ considerably in their calcium content. This may be important to researchers employing albumin in situations where calcium concentration is critical.

BDM also has strong protective effects in our experimental system. Thus, FBP and BDM may be useful in hypothermic preservation of hearts for transplantation. Because calcium levels are normally well controlled in vivo, especially in clinical situations, calcium chelation might be less relevant compared to other beneficial effects of these two agents. However, during ex vivo preservation experiments, this could be a major factor in cardiomyocyte survival. Pyruvate, adenine, and ribose had little or no beneficial effects during the ischemic, hypothermic incubation. However, it remains possible that these compounds could be protective upon return to physiological temperature and oxygen levels.
